# Impact of field‐realistic doses of glyphosate and nutritional stress on mosquito life history traits and susceptibility to malaria parasite infection

**DOI:** 10.1002/ece3.6261

**Published:** 2020-04-27

**Authors:** Danaé Bataillard, Philippe Christe, Romain Pigeault

**Affiliations:** ^1^ Department of Ecology and Evolution University of Lausanne Lausanne Switzerland

**Keywords:** diet, glyphosate‐based herbicides, *Plasmodium relictum*, vector

## Abstract

Glyphosate is the world's most widely used herbicide. The commercial success of this molecule is due to its nonselectivity and its action, which would supposedly target specific biosynthetic pathways found mainly in plants. Multiple studies have however provided evidence for high sensitivity of many nontarget species to glyphosate and/or to formulations (glyphosate mixed with surfactants). This herbicide, found at significant levels in aquatic systems through surface runoffs, impacts life history traits and immune parameters of several aquatic invertebrates' species, including disease‐vector mosquitoes. Mosquitoes, from hatching to emergence, are exposed to aquatic chemical contaminants. In this study, we first compared the toxicity of pure glyphosate to the toxicity of glyphosate‐based formulations for the main vector of avian malaria in Europe, *Culex pipiens* mosquito. Then we evaluated, for the first time, how field‐realistic dose of glyphosate interacts with larval nutritional stress to alter mosquito life history traits and susceptibility to avian malaria parasite infection. Our results show that exposure of larvae to field‐realistic doses of glyphosate, pure or in formulation, did not affect larval survival rate, adult size, and female fecundity. One of our two experimental blocks showed, however, that exposure to glyphosate decreased development time and reduced mosquito infection probability by malaria parasite. Interestingly, the effect on malaria infection was lost when the larvae were also subjected to a nutritional stress, probably due to a lower ingestion of glyphosate.

## Graphical abstract

Glyphosate is the world's most widely used herbicide. The commercial success of this molecule is due to its nonselectivity and its action, which would supposedly target specific biosynthetic pathways found mainly in plants. However, we have shown that exposure to field‐realistic dose of glyphosate decreases the development time of mosquito larvae and reduces mosquito infection probability by malaria parasite. Interestingly, the effect on malaria infection was lost when the larvae are subjected to a nutritional stress, probably due to a lower ingestion of glyphosate.

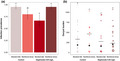

## 1 INTRODUCTION

Glyphosate is the world's most widely used herbicide. More than 85,000 tons are spread every year (Benbrook, 2016). The commercial success of glyphosate is due to its nonselectivity and its action, which would supposedly target specific biosynthetic pathways found only in plants and certain microorganisms, thus minimizing its toxicity to nontarget organisms. Many studies belie this assumption and provided evidence for high sensitivity of many nontarget species to glyphosate (Annett, Habibi, & Hontela, 2014; Gill, Sethi, Mohan, Datta, & Girdhar, 2018). In addition, glyphosate is mixed with surfactants to improve the performance of this foliar‐applied herbicide (surfactants increase the surface area in contact with the vegetation). Comparative toxicity assessments between formulations (glyphosate mixed with surfactants) and pure glyphosate show that formulations are more toxic than glyphosate alone (Gill et al., 2018; Nagy et al., 2019 but see Pochron et al., 2020).

It has been estimated that less than 0.1% of glyphosate‐based herbicides applied to crops reach their specific targets (Nguyen, Nguyen, Hwang, Bui, & Park, 2016). Herbicide and their degradation products are therefore found at significant levels in the environment (Bai & Ogbourne, 2016; Peruzzo, Porta, & Ronco, 2008; Struger et al., 2008) and, in particular, in aquatic systems through surface runoffs (Giesy, Dobson, & Solomon, 2000; Morrissey et al., 2015; Van Bruggen et al., 2018). Exposure to sublethal concentrations of glyphosate, pure or in formulation, induces biochemical, physiological, and behavioral alterations in many fish and amphibian species (Daam, Moutinho, Espíndola, & Schiesari, 2019; Langiano & Martinez, 2008; Mann & Bidwell, 1999; Modesto & Martinez, 2010). An increasing number of studies also showed negative effects of glyphosate exposure on life history traits (Hansen & Roslev, 2016; Janssens & Stoks, 2017; Kibuthu, Njenga, Mbugua, & Muturi, 2016; Morris, Murrell, Klein, & Noden, 2016) and immune parameters (Matozzo, Zampieri, Munari, & Marin, 2019; Mohamed, 2011; Monte et al., 2019) of several aquatic invertebrates' species, including disease‐vector mosquitoes. Mosquitoes are the most dominant group of bloodsucking Diptera responsible for transmission of numerous viral, bacterial, and parasitic diseases of humans and other vertebrates.

The preimaginal stages of mosquitoes develop in freshwater environments and, from hatching to emergence, larvae and pupae may therefore be exposed to aquatic chemical contaminants. Glyphosate exposure may alter mosquito behavior (Baglan, Lazzari, & Guerrieri, 2018), life history traits such as larval survival rate or reproduction (Bara, Montgomery, & Muturi, 2014; Kibuthu et al., 2016; Morris et al., 2016 but see Baglan et al., 2018), as well as the expression of genes conferring resistance to insecticides (Riaz et al., 2009). Glyphosate exposure has therefore the potential to influence the vectorial capacity of mosquitoes. To date, no studies have however tested whether exposure to glyphosate could directly affect mosquito susceptibility to vector‐borne parasite infections and whether this potential impact is modulated by larval nutritional stress. In the wild, mosquito larvae are exposed simultaneously to multiple biotic (competition, predation) and abiotic (temperature, bioactive contaminants, food quality, Beketov & Liess, 2007; Muturi, Kim, Alto, Berenbaum, & Schuler, 2011; Tripet, Aboagye‐Antwi, & Hurd, 2008) stresses. All possible outcomes of stressor combinations (i.e., additive, synergistic, or antagonistic) may be observed (Coors & Meester, 2008; Crain, Kroeker, & Halpern, 2008) and influence vector‐borne pathogen transmission directly through changes in vector density or indirectly by changing vector immunocompetence, lifespan, or reproductive potential.

Our specific aims in this study were to answer three questions. First, does exposure of larvae to glyphosate influence mosquito life history traits and susceptibility to malaria parasite infections? Second, is the potential effect of glyphosate‐based herbicides (formulation) stronger than glyphosate alone? Third, is there an additive, synergistic, or antagonistic effect between two different stressors, namely glyphosate exposure and food limitation? Experiments were conducted with a natural system consisting of the avian malaria parasite *Plasmodium relictum* and its vector in the wild, the mosquito *Culex pipiens* (Pigeault et al., 2015).

## 2 MATERIALS AND METHODS

### 2.1 Malaria parasites

*Plasmodium relictum* (lineage SGS1) is the most prevalent agent of avian malaria in Europe (Valkiunas, 2004). The parasite strain used in the first experiment was isolated from an infected Great tit (*Parus major*) in Lausanne, Switzerland in June 2018. Since then, the parasite has been maintained by carrying out two passages across a stock canary (*Serinus canaria*) through intraperitoneal injections (i.p) until the experiment (November 2018, Pigeault et al., 2015). The *Plasmodium relictum* strain (lineage SGS1) used in the second experiment was isolated two months before the experiment (January 2019) from an infected House sparrow (*Passer domesticus*) captured in the field and passaged once by i.p. to naïve canaries prior to the experiment. *Plasmodium* lineage (SGS1) used in this study was identified from blood samples using molecular methods. Briefly, a nested PCR (Hellgren, Waldenström, & Bensch, 2004) was performed on samples after DNA was extracted from blood using a DNeasy Blood & Tissue Kit (Qiagen) according to the manufacturer's instructions. Nested PCR products were sequenced as in Rooyen, Lalubin, Glaizot, and Christe (2013) and identified by performing a local BLAST search in the MalAvi database (http://mbio‐serv2.mbioekol.lu.se/Malavi/, Bensch, Hellgren, & Pérez‐Tris, 2009).

Prior to the experimental infections, a small amount (ca. 3–5 µl) of blood was collected from the medial metatarsal vein of each canary to ensure that they were free from any previous hemosporidian infections. Three and four birds, for the experiment one and two, respectively, were then inoculated by i.p injection of 100 µl of an infected blood solution. Blood solution was constituted with a mixture of PBS and blood (ratio 1:1) sampled from three canaries infected with the parasite three weeks before the experiment. Ten days postinfection, bird parasitemia (percentage of infected red blood cell) was estimated by visual quantification on blood smears (Valkiunas, 2004).

### 2.2 Mosquito rearing and experimental infections

Both experiments were conducted with a lineage of *Culex pipiens* mosquitoes, the main vector of *Plasmodium relictum* in Europe, collected in the field (Lausanne, Switzerland) and maintained in insectary under standard conditions (26 ± 1°C, 70 ± 5% RH and 12L: 12D photoperiod) since August 2017. The mosquito species was identified by morphology in the laboratory (Schaffner et al., 2001). For each experiment, mosquito egg rafts were obtained by feeding 30–40 females on one healthy bird (*Serinus canaria*, the bird used was different for experiments 1 and 2). Eggs were then placed in the same plastic tank. One day posthatching, larvae were randomly collected and assigned to the different treatment groups (see below). Larvae were reared individually in plastic tube (30 ml) filled with 6 ml of solution (see below). Larval mortality and development time were daily recorded until adult emergence. Immediately after emergence, mosquitoes belonging to the same treatment were placed inside an adult rearing cage. Males and females were kept together and fed ad libitum on a 10% glucose water solution during 5 ± 2 days.

Afterwards, 15 ± 2 or 10 ± 2 females, for the experiments 1 and 2, respectively, from each experimental treatment were placed together inside a feeding cage (2 and 3 different cages for experiments 1 and 2, respectively) and allowed to feed from a *Plasmodium*‐infected bird for 3 hr. To minimize host defensive behaviors that may alter the mosquito biting process during the assay, birds were immobilized as described in Cornet, Nicot, Rivero, & Gandon, (2013). All engorged females were then taken out from the cages, briefly anesthetized with CO2, counted, and placed individually into numbered dry 30‐ml plastic tubes covered with a mesh. Food was provided in the form of a cotton pad soaked in a 10% glucose solution placed on top of each tube. To distinguish between the treatments, mosquitoes were previously marked using different fluorescent color powders and the color allocated to each treatment changed in each of the cages (for details see Vézilier, Nicot, Gandon, & Rivero, 2010). Five days postblood meal, females were taken out of the tubes and placed in new plastic tubes filled with 4 ml of water to encourage them to lay eggs. The amount of hematin excreted at the bottom of each first tube was quantified as an estimate of the blood meal size (Vézilier et al., 2010). Three days later (day 8 postblood meal), females were taken out of the tubes. The egg rafts were collected and the number of eggs was counted under a binocular microscope. One wing was removed from each female and measured under a binocular microscope along its longest axis as an index of body size (Van Handel & Day, 1989). Females were then dissected, and the number of *Plasmodium* parasite (oocyst stage) present in their midguts were counted under a binocular microscope. The wing size was also measured for all males.

### 2.3 Glyphosate exposure

#### 2.3.1 Experiment 1: pure glyphosate and glyphosate‐based herbicides

The purpose of this experiment was to test the impact of pure glyphosate and glyphosate‐based herbicides (formulation), on mosquito life history traits and susceptibility to malaria parasite infection. For this purpose, one‐day‐old mosquito larvae were randomly assigned to five experimental groups (120 larvae per group). Two groups corresponded to 2 different concentrations of pure glyphosate (0.05 and 0.1 mg/L), and two groups corresponded to the same 2 concentrations of glyphosate but in formulation. The remaining group was reared without glyphosate (control). Larvae were fed daily with 0.5 mg of food, which is consistent with our standard colony maintenance diet (1:1 fish food and rabbit pellets). The concentrations of glyphosate used in the experiment 1 (0.05 and 0.1 mg/L) were chosen based on concentrations observed in aquatic systems (see table 10 in Giesy et al., 2000, Silva et al., 2018) as well as in previous studies on the impact of sublethal doses of pesticides on aquatic invertebrates species (Baglan et al., 2018; Kibuthu et al., 2016; Riaz et al., 2009). For pure glyphosate solution, rearing solutions were prepared from a stock solution of 2 mg/L produced with solid glyphosate (96% purity; provided by Sigma‐Aldrich) dissolved in mineral water. From the stock solution, 2 derived solutions were prepared (0.05 and 0.1 mg/L). For the glyphosate‐based herbicides, a formulated glyphosate solution (glyphosate concentration: 360 g/L; Sintagro®) was diluted ten times with mineral water to obtain a stock solution (glyphosate concentration: 3.60 g/L). From this stock solution, 2 derived solutions were prepared to obtain the two glyphosate concentrations: 0.05 and 0.1 mg/L.

Stock solutions were renewed every 6 days, to avoid any degradation effect (Baglan et al., 2018). Rearing solution was replaced every 3 days to ensure that the larvae were exposed to the same concentration of glyphosate during all their development.

#### 2.3.2 Experiment 2: pure glyphosate and food limitation

The purpose of the second experiment was to evaluate the impact of larval exposure to glyphosate associated with a nutritional stress on mosquito life history traits and susceptibility to malaria parasite infection. For this purpose, larvae, reared in a 0.05 mg/L pure glyphosate solution, were either daily fed with 0.5 mg (standard colony maintenance diet) or with 0.25 mg of food (nutritional stress). The specific food treatments were chosen based on a pilot study, with the goal of generating a nutritional stress that did not significantly impact larval survival rate. One‐day‐old mosquito larvae were randomly assigned to four experimental groups (160 larvae per group): (a) glyphosate exposure and standard diet, (b) glyphosate exposure and nutritional stress, (c) unexposed to glyphosate and standard diet, and (d) unexposed to glyphosate and nutritional stress. Glyphosate solution was prepared as described above with solid glyphosate (96% purity; provided by Sigma‐Aldrich).

### 2.4 Statistical analysis

All statistical analyses were performed with R (version 3.4.1). The sample sizes included in each analysis and the different statistical models built to analyze the data are described in the supplementary material (Table S1 and S2). Larval survival rate was analyzed using Cox proportional hazards regression model (coxph, survival package). Explanatory variables were glyphosate concentration and glyphosate type (pure or in formulation) for the experiment 1 and glyphosate exposition and food treatments for the experiment 2. A GLM with normal distribution of errors was used to test for difference in development time (calculated as the number of days from hatching to emergence) and wing length (mm) among the larval treatments. Explanatory variables were mosquito sex, glyphosate concentration, and glyphosate type (pure or in formulation) for the experiment 1 and mosquito sex, glyphosate exposition, and food treatments for the experiment 2. Female mosquito‐centered traits (proportion of females which took a blood meal, blood meal size, number of eggs, infection prevalence, and oocyst burden), which may depend on which bird mosquitoes fed on, were analyzed fitting bird as a random factor into the models (to account for the spatial pseudoreplication), using *lmer* or *glmer* (package: lme4, Bates, Mächler, Bolker, & Walker, 2015) according to whether the errors were normally (blood meal size, number of eggs, and oocyst burden) or binomially (proportion of females which took a blood meal, prevalence) distributed. Glyphosate concentration and glyphosate type (pure or in formulation) for the experiment 1 and glyphosate exposition and food treatments for the experiment 2 were used as fixed factors. Blood meal size was also added as a fixed factor (continuous variable) when it was not a response variable.

Maximal models, including all higher‐order interactions, were simplified by sequentially eliminating nonsignificant interactions and terms to establish a minimal model (Crawley, 2012). The significance of the explanatory variables was established using either a likelihood ratio test (which is approximately distributed as a chi‐square distribution, Bolker, 2008) or an *F* test. The significant chi‐square or *F* values given in the text are for the minimal model, whereas nonsignificant values correspond to those obtained before the deletion of the variable from the model. A posteriori contrasts were carried out by aggregating factor levels together and by testing the fit of the simplified model using LRT (Crawley, 2012).

## 3 RESULTS

### 3.1 Experiment 1: pure glyphosate versus glyphosate‐based herbicides

There was no effect of glyphosate type (pure or formulation) on larval mortality (model 1: $\chi_{1}^{2}$ = 1.23, *p* = .267), but glyphosate concentration (0, 0.05 and 0.1 mg/L) had a significant impact (model 1: $\chi_{1}^{2}$ = 6.21, *p* = .013). Contrast analyses showed that larval mortality was higher in mosquitoes reared in 0.05 mg/L glyphosate solution than in mosquitoes reared in 0.1 mg/L glyphosate solution or without glyphosate (contrast analyses: 0.05 mg/0.1 mg: $\chi_{1}^{2}$ = 6.33, *p* = .012, 0.05 mg/0 mg: $\chi_{1}^{2}$ = 4.12, *p* = .042, larval survival rate: 0 mg/L: 0.89, 95% IC 0.82–0.93, 0.05 mg/L: 0.81, 95% IC 0.76–0.86 and 0.1 mg/L: 0.89, 95% IC 0.85–0.93). No difference was observed between control larvae (0 mg/L) and larvae reared in 0.1 mg/L glyphosate solution (contrast analysis: $\chi_{1}^{2}$ = 0.01, *p* = .98).

Glyphosate concentration and glyphosate type did not have significant effect on both larval development time (model 2: glyphosate concentration: *F* = 1.93, *p* = .165, glyphosate type: *F* = 1.87, *p* = .154) and adult mosquito size (model 3: glyphosate concentration: *F* = 0.12, *p* = .732, glyphosate type: *F* = 2.08, *p* = .150). As expected in mosquitoes an effect of sex was observed, males had a shorter development time (model 2: *F* = 629.38, *p* < .0001, mean ± s.e., male: 7.67 ± 0.03 days, female: 9.66 ± 0.07 days) and were smaller than females (model 3: *F* = 314.16, *p* < .0001).

Glyphosate concentration and glyphosate type did not have significant effect on the proportion of females which took a blood meal (model 4: glyphosate concentration: $\chi_{1}^{2}$ = 2.56, *p* = .109, glyphosate type: $\chi_{1}^{2}$ = 0.45, *p* = .50, blood meal rate = 0.84, 95% IC: 0.79–0.91). While larvae exposed to the highest concentration of glyphosate (0.1 mg/L) tended to take a smaller blood meal (0 mg/L: 28.6 µg ± 1.9, 0.05 mg/L: 29.6 µg ± 1.4, 0.1 mg/L: 26.7 µg ± 1.1), the amount of blood ingested did not vary significantly between treatments (model 5: glyphosate concentration: $\chi_{1}^{2}$ = 2.76, *p* = .09; glyphosate type $\chi_{1}^{2}$ = 0.12, *p* = .724). No effect of glyphosate concentration and glyphosate type was observed on the number of laid eggs (model 6: $\chi_{1}^{2}$ = 2.19, *p* = .139; $\chi_{1}^{2}$ = 0.40, *p* = .526, respectively). A positive relationship was observed between blood meal size and the number of laid eggs (model 6: $\chi_{1}^{2}$ = 24.39, *p* < .0001).

Midgut dissection revealed that 100% of the mosquitoes fed on infected bird blood were infected with *Plasmodium relictum.* Glyphosate concentration and glyphosate type had no effect on the oocyst burden of mosquitoes (model 7: $\chi_{1}^{2}$ = 0.11, *p* = .895, $\chi_{1}^{2}$ = 0.02, *p* = .895, respectively). A positive relationship was observed between blood meal size and oocyst burden (model 7: $\chi_{1}^{2}$ = 25.56, *p* < .0001).

### 3.2 Experiment 2: glyphosate and nutritional stress

**FIGURE 1:**
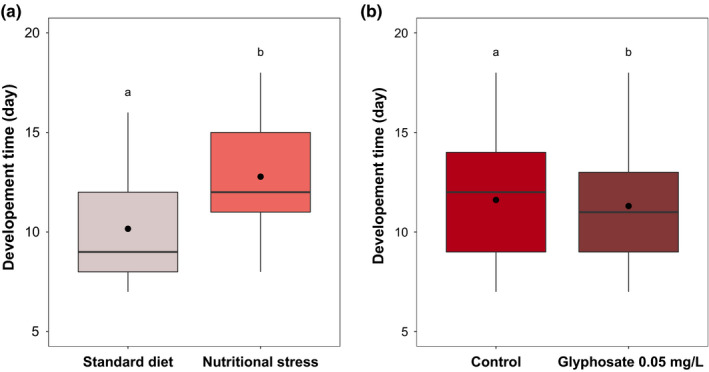
Impact of (a) food treatment and (b) glyphosate exposure on larval development time. Boxplots were constructed to show the raw data. Boxes above and below the medians (horizontal lines) show the first and third quartiles, respectively. Black points represent the means. Levels not connected by the same letter are significantly different (*p* < .05)

Glyphosate exposure (0.05 mg/L) and food treatments had no significant effect on larval mortality (model 8: $\chi_{1}^{2}$ = 3.83, *p* = .067, $\chi_{1}^{2}$ = 0.01, *p* = .961, respectively). Variation in larvae development time was explained by food treatment, glyphosate exposure, and mosquito sex (model 9: *F* = 366.08, *p* < .0001, *F* = 8.123, *p* = .004, *F* = 655.21, *p* < .0001, respectively). While nutritional stress impacted negatively the development time (Figure 1a), larvae exposed to glyphosate developed faster than unexposed larvae (Figure 1b). As expected, males had a shorter development time than females (male: 9.9 ± 0.1 days, female: 13.4 ± 0.2).

Food treatments and sex, but not glyphosate exposure, impacted mosquito size (model 10: *F* = 39.20, *p* < .0001, *F* = 384.85, *p* < .0001, *F* = 2.06, *p* = .153, respectively). Males were smaller than females, and adults from larvae reared under standard diet condition were bigger than adults from larvae reared under nutritional stress (mean ± s.e. optimal feeding condition = 0.30 ± 0.003, food limitation = 0.28 ± 0.003).

Glyphosate concentration and glyphosate type did not have significant effect on the proportion of females which took a blood meal (model 11: glyphosate exposure: $\chi_{1}^{2}$ = 1.54, *p* = .214, food treatments: $\chi_{1}^{2}$ = 0.01, *p* = .908, blood meal rate = 0.80, 95% IC: 0.72–0.88). The amount of blood ingested by females as well as the number of laid eggs did not vary between treatments (blood meal size: model 12: glyphosate exposure = $\chi_{1}^{2}$ = 1.27, *p* = .259; food treatments = $\chi_{1}^{2}$ = 1.29, *p* = .256; eggs number: model 13: glyphosate exposure = $\chi_{1}^{2}$ = 0.01, *p* = .99; food treatment = $\chi_{1}^{2}$ = 3.49, *p* = .066). A positive relationship was observed between blood meal size and the number of laid eggs (model 13: $\chi_{1}^{2}$ = 5.70, *p* = .017).

**FIGURE 2:**
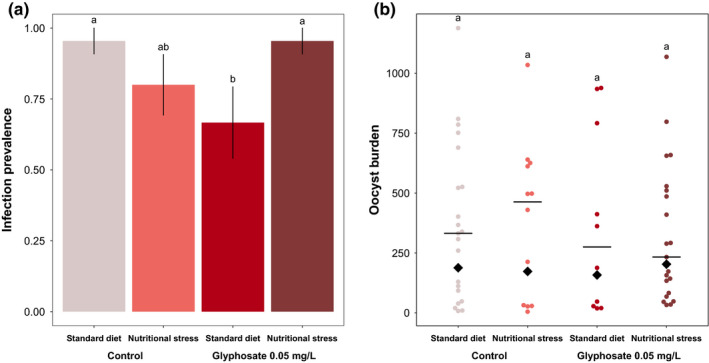
Impact of food treatment and glyphosate exposure on (a) the probability of mosquitoes to be infected by malaria parasites and on (b) the intensity of infection (oocyst burden). In panel (b) black horizontal lines represent medians, and black diamond represents means. Levels not connected by the same letter are significantly different (*p* < .05)

Significant interaction of glyphosate exposure and food treatments was observed regarding the probability of females to be infected by malaria parasites (model 14: $\chi_{1}^{2}$ = 7.67, *p* = .006, Figure 2a). In the absence of glyphosate, nutritional stress tended to decrease the infection prevalence (infection prevalence = nutritional stress: 0.80, 95% IC 0.54–0.92, standard diet: 0.95, 95% IC 0.78–0.99; contrast analysis: $\chi_{1}^{2}$ = 2.74, *p* = .097). However, in the presence of glyphosate, the infection prevalence observed in females from larvae reared with standard diet was roughly a third lower than that of females from the nutritional stress treatment (infection prevalence = nutritional stress: 0.95, 95% IC 0.78–0.99, standard diet condition: 0.66, 95% IC 0.41–0.84; contrast analysis: $\chi_{1}^{2}$ = 5.18, *p* = .022). It is also interesting to note that when larvae are reared without nutritional stress, the infection prevalence observed in mosquitoes exposed to glyphosate was significantly lower than in unexposed mosquitoes (contrast analysis: $\chi_{1}^{2}$ = 5.37, *p* = .020, Figure 2a). Infection prevalence tended to but was not significantly impacted by blood meal size (model 14: $\chi_{1}^{2}$ = 3.13, *p* = .076).

The intensity of the infection was not impacted by glyphosate exposure, food treatments, or by the interaction between the two factors (model 15: $\chi_{1}^{2}$ = 0.56, *p* = .455, $\chi_{1}^{2}$ = 0.98, *p* = .321, $\chi_{1}^{2}$ = 0.08, *p* = .782, respectively, Figure 2b), but a positive relationship was observed between blood meal size and oocyst burden (model 15: $\chi_{1}^{2}$ = 13.97, *p* = .0001).

## 4 DISCUSSION

In this study, we assessed the consequences of larval exposure to pure glyphosate or glyphosate‐based herbicide on *Culex pipiens* mosquito life history traits and susceptibility to avian malaria parasite infection. While we did not observe significant effect of glyphosate on mosquito life history traits, we found that this compound reduced the prevalence of *Plasmodium* parasite infection under standard diet. Interestingly, this effect was lost when the larvae were subjected to nutritional stress.

### 4.1 Glyphosate exposure and mosquito life history traits

Exposure of larvae to the two realistic concentrations of glyphosate had no significant effect on mosquito size and other life history traits. Although our second experiment tended to show a slight effect of glyphosate (0.05 mg/L) on larval development time, this was not observed in the first experiment even when the concentration of glyphosate in the rearing solution was doubled. These results are consistent with a recent study that found no effect of glyphosate on *Aedes aegypti* mosquito larvae at concentrations 20 times higher than ours (Baglan et al., 2018). In a pilot study, we even showed that a glyphosate concentration of 1 g/L did not affect larval survival rate. In other aquatic invertebrate species, this molecule tends to have more significant effect. Glyphosate exposure, even at low concentration (0.1–0.05 mg/L), induced a reduction of juvenile size in the planktonic crustacean *Daphnia magna* (Cuhra, Traavik, & Bøhn, 2013) and negatively impacted the survival rate in the amphipod *Hyalella castroi* and the crayfish *Cherax quadrinatus* (Avigliano, Fassiano, Medesani, Ríos de Molina, & Rodríguez, 2014; Dutra, Fernandes, Failace, & Oliveira, 2011). This suggests that mosquito larvae are more tolerant to glyphosate than other aquatic invertebrate species. There are, nonetheless, other parameters that may modulate the negative impact of glyphosate on invertebrate life history traits and could therefore explain our results. A study on *Anopheles arabiensis* described that the insecticide resistance phenotypes may interfere with mosquito response to glyphosate exposure (Oliver & Brooke, 2018). However, although the insecticide resistance phenotype of our mosquito lineage is unknown, we found that it was highly susceptible to the best‐known and most widely used representative of neonicotinoid insecticides (imidacloprid, Figure S1).

### 4.2 Glyphosate exposure and mosquito susceptibility to *Plasmodium* infection

The impact of glyphosate on the susceptibility of mosquitoes to malaria parasite infection is unclear. While the probability of infection of females was 100% in the first experiment, we found that exposure to glyphosate in the larval stage reduced the prevalence of *Plasmodium* infection by roughly one‐third in the second experiment. Fluctuations in the prevalence of infection between experimental blocks are common (Pigeault et al., 2015). This might be due to the difference in parasite strains and parasite loads in infected canaries between the experiments. The low sample sizes used to evaluate infection prevalence may also explain the difference between the two experiments (Table S1). Nevertheless, the observed effects in the second experiment may be due to an effect of glyphosate on mosquito immunity. Glyphosate can affect invertebrate immunity in several ways, including effects on hemocyte parameters (Hong, Yang, Huang, Yan, & Cheng, 2018; Hong et al., 2017; Matozzo et al., 2019; Monte et al., 2019), activity of immune enzymes (Hong et al., 2017), and on oxidative stress (de Melo Tarouco et al., 2017; Pala, 2019). The effects on invertebrate immunity were generally negative. For instance, the exposure of shrimps (*Macrobrachium nipponensis*), Chinese mitten crabs (*Eriocheir sinensisto*), and clams (*Ruditapes philippinarumto*) to a sublethal concentrations of glyphosate caused a significant decrease in total hemocyte count (Hong et al., 2017, 2018; Matozzo et al., 2019). However, in the clam *R. philippinarum*, exposure to glyphosate increased significantly hemocyte proliferation and both diameter and volume of these immune cells (Matozzo et al., 2019). In the Chinese mitten crabs, high concentrations of glyphosate increased the phenoloxidase (PO) activities. Knowing that the phenoloxidase cascade is an important immune response of mosquitoes that leads to the encapsulation and death of various parasites, including *Plasmodium*, (Christensen, Li, Chen, & Nappi, 2005; Yassine, Kamareddine, & Osta, 2012; Zhang et al., 2008) an effect of glyphosate on this immune parameter could have a positive impact on mosquito immunocompetence.

Another nonexclusive reason that could explain lower prevalence of infection in mosquitoes exposed to glyphosate would be the effect of this molecule on their midgut (Gregorc & Ellis, 2011). Some other pesticides, such as imidacloprid, disrupt the development of mosquitoes' midgut (Fernandes et al., 2015). Imidacloprid significantly reduced the number of digestive and endocrine cells, resulting in malformation of the midgut epithelium (Fernandes et al., 2015). The midgut epithelial membrane is the first barrier that parasites must cross to infect their vector. Although a malformation of the midgut may reduce the ability of the parasite to cross the epithelial membrane, the complete opposite effect could be also expected. Indeed, the midgut disruption could lead to a less efficient physical barrier favoring infection. Mosquito midgut produces also a large amount of xanthurenic acid which is essential for the exflagellation of *Plasmodium* transmissible stages (gametocytes, Billker et al., 1998), and the secretion of digestive carboxypeptidase enzymes may also provide parasite with essential amino acids for its development (Lavazec & Bourgouin, 2008). An alteration of the midgut could therefore impact the parasite's ability to invade this organ. It is important to note that while exposure to glyphosate did not affect the size of adult mosquitoes, females derived from larvae exposed to the highest concentrations of glyphosate (0.1 mg/L) tended to excrete less hematin. This result may indicate an effect of glyphosate on their ability to digest blood potentially due to a midgut malformation.

While we observed an effect of glyphosate exposure on the prevalence of *Plasmodium* infection, no effect on the oocyst burden was observed. By decomposing the infection phenotype into infection prevalence and intensity, a study had shown that some genes underlying variation in infection intensity may be distinct from those controlling variation in infection prevalence (Harris et al., 2010). Different mechanisms, involving different pathways (e.g., microbiota, epithelial immunity), may therefore control the different steps of the infection process. Here, if larval exposure to glyphosate impacts the early stages of parasite development in mosquito midgut or the rate of fertilization of the gametes (e.g., by stimulating mosquito immune system or by modifying the secretory capacity of the midgut), then mosquitoes that have ingested only a few gametocytes may not become infected by the parasite. However, the effect of exposure to a low dose of glyphosate may not be sufficient to block the development and therefore infection by *Plasmodium* when the quantity of parasites ingested exceeds a certain threshold.

### 4.3 Glyphosate exposure and larval nutritional stress

Many natural (e.g., competition, predation, temperature, nutritional stress) and human‐induced stressors (e.g., chemical contaminant) are known to impact negatively mosquito life history traits and immunity (Martínez‐De la Puente, Gutierrez‐López, & Figuerola, 2018; Muturi, Nyakeriga, & Blackshear, 2012; Shapiro, Murdock, Jacobs, Thomas, & Thomas, 2016; Vantaux et al., 2016). In nature, mosquito larvae are exposed to multiple stressors and, while all possible outcomes of stressor combinations (i.e., additive, synergistic, or antagonistic) may be expected, the effect of the interaction between stressors is overlooked in studies on mosquito–parasite interaction. To our knowledge, this is the first study to evaluate how field‐realistic dose of glyphosate interacts with larval nutritional stress to alter mosquito life history traits and susceptibility to malaria parasite infection. As expected, nutritional stress resulted in longer development time and smaller‐sized adults (Araújo, Gil, & e‐Silva, 2012; Takken et al., 2013; Vantaux et al., 2016). The only interaction we observed between food treatment and glyphosate exposure was on the probability of infection of females. The addition of nutritional stress during larval development appeared to have alleviated the positive effect of glyphosate on the prevalence of *Plasmodium* infection. When larvae were exposed to glyphosate, the prevalence of infection was significantly higher when a nutritional stress was applied. The amount of glyphosate ingested by the larvae should increase in proportion to the amount of food ingested. Assuming that glyphosate has an effect on the midgut physiology (Gregorc & Ellis, 2011), if the larvae have less food, they will ingest less glyphosate, which will reduce the effect of this molecule.

### 4.4 Pure glyphosate versus glyphosate‐based herbicide

We also investigated whether a glyphosate‐based herbicide was more harmful than glyphosate alone. Numerous studies have demonstrated that glyphosate formulations (glyphosate mixed with surfactants) are more toxic than pure glyphosate (reviewed in Nagy et al., 2019) but our study did not show any difference. However, the majority of these studies used Roundup® formulation which is described as the most cytotoxic herbicide (Mesnage, Defarge, Spiroux de Vendômois, & Séralini, 2014; Nagy et al., 2019). The surfactants associated with glyphosate vary from one formulation to another. Here, we used the Sintagro® formulation and our results suggest that the surfactant included in this formulation (i.e., alkyl polyglucoside), which is different from that contained in Roundup® (i.e., polyoxyethyleneamine), had no effect, at the concentrations used in our study, on mosquito's life history traits. While the surfactant polyoxyethyleneamine facilitates glyphosate penetration through plasmatic membranes and consequently makes formulations more toxic than glyphosate alone (but see Pochron et al., 2020), alkyl polyglucoside is generally considered safe and environmentally friendly because of their low toxicity and high biodegradability (Rybinski & Hill, 1998).

## 5 CONCLUSION

Our results show that exposure to field‐realistic doses of glyphosate at larval stages, pure or in formulation, did not affect mosquito larval survival rate, adult size, and female fecundity. One of our two experimental blocks showed, however, an effect of glyphosate on the development time and on the probability of female infection by *Plasmodium* parasite. However, the effect on infection prevalence was lost when the larvae are subjected to a nutritional stress, probably due to a lower ingestion of glyphosate. Altogether, our study and those published recently tend to suggest that mosquito larvae are more tolerant to glyphosate than many other invertebrate species. It has been recently shown that adult exposure to glyphosate perturbs the gut microbiota of honey bees and ultimately their susceptibility to parasite infection (Motta, Raymann, & Moran, 2018). It would be therefore relevant to also investigate the effect of glyphosate exposure at the adult stage on mosquito susceptibility to malaria parasite infection. Another important factor that would be relevant to consider is the effect of glyphosate exposure, at larval and/or adult stage, on mosquito longevity. The lifespan of mosquitoes has drastic consequences for the *Plasmodium* transmission success (e.g., increases the potential for infective bites to hosts, Smith & Ellis McKenzie, 2004).

## AUTHOR CONTRIBUTIONS

**Danaé Bataillard**: Conceptualization (equal); Formal analysis (equal); Investigation (lead); Methodology (equal). **Philippe Christe**: Conceptualization (equal); Funding acquisition (equal); Supervision (equal); Validation (equal); Writing‐review & editing (equal). **Romain Pigeault**: Conceptualization (equal); Formal analysis (equal); Methodology (equal); Supervision (equal); Validation (equal); Writing‐original draft (lead); Writing‐review & editing (equal).

## COMPETING INTERESTS

The authors declare that they have no competing interests.

## ETHICAL APPROVAL

This study was approved by the Ethical Committee of the Vaud Canton veterinary authority, authorization number 1730.4.

### Open Research Badges

This article has earned an Open Data Badge for making publicly available the digitally‐shareable data necessary to reproduce the reported results. The data is available at https://doi.org/10.5061/dryad.xgxd254cw.

## Supporting information

Supplementary Material

## Data Availability

All data supporting the conclusions of this paper are available on the Dryad website: https://doi.org/10.5061/dryad.xgxd254cw.
