# Supplementary material for: Impact of field‐realistic doses of glyphosate and nutritional stress on mosquito life history traits and susceptibility to malaria parasite infection
Source: Ecol Evol. 2020 Apr 27;10(11):5079–88. doi: 10.1002/ece3.6261 (PMC7297737; doi:10.1002/ece3.6261)
Supplement: Supplementary file 1 — Supplementary Material [file ECE3-10-5079-s001.docx]

**Impact of field-realistic doses of glyphosate and nutritional stress on mosquito life history-traits and susceptibility to malaria parasite infection**

Danaé Bataillard^1^, Philippe Christe^1^, Romain Pigeault^1^*

1 Department of Ecology and Evolution, CH-1015 Lausanne, Switzerland

*Corresponding author: romain.pigeault@unil.ch

**ORCIDs:**

R. Pigeault: 0000-0002-8011-4600

P. Christe: 0000-0002-8605-7002

**Supplementary information:**

**Table S1**

**Table S2**

**Figure S1**

|  |  | Glyphosate concentration: 0.05 mg/L | | Glyphosate concentration: 0.1 mg/L | | 0 mg/L |  |
| --- | --- | --- | --- | --- | --- | --- | --- |
|  |  | Pure | Formulation | Pure | Formulation |  | **Total** |
| **Experiment 1** | Survival rate | 116 | 118 | 113 | 120 | 119 | 586 |
|  | Dev time | 91 | 99 | 100 | 108 | 106 | 504 |
|  | Wing size | 53 | 49 | 50 | 62 | 55 | 269 |
|  | Blood meal rate | 30 | 30 | 30 | 30 | 30 | 150 |
|  | Blood meal size | 25 | 20 | 24 | 26 | 24 | 119 |
|  | Number of eggs | 18 | 14 | 14 | 14 | 14 | 74 |
|  | Oocyst burden | 21 | 20 | 19 | 22 | 22 | 104 |

**Supplementary Table S1.**  **Sample size included in each analysis**

|  |  | Glyphosate concentration: 0 mg/L | | Glyphosate concentration: 0.05 mg/L | |  |
| --- | --- | --- | --- | --- | --- | --- |
|  |  | Nutritional stress | Standard diet | Nutritional stress | Standard diet | **Total** |
| **Experiment 2** | Survival rate | 152 | 158 | 153 | 155 | 618 |
|  | Dev time | 109 | 104 | 110 | 122 | 445 |
|  | Wing size | 79 | 77 | 79 | 77 | 312 |
|  | Blood meal rate | 30 | 32 | 34 | 24 | 120 |
|  | Blood meal size | 21 | 28 | 31 | 19 | 99 |
|  | Number of eggs | 5 | 12 | 3 | 5 | 25 |
|  | Infection prevalence | 15 | 22 | 22 | 15 | 74 |
|  | Oocyst burden | 12 | 21 | 21 | 10 | 64 |

**Supplementary Table S2.** Description of statistical models used in the study. N gives the number of mosquitoes included in each analysis. "Maximal model" gives the complete set of explanatory variables included in the model. "Minimal model" gives the model containing only the significant variables and their interactions. Parentheses indicate variables fitted as random factors. Square brackets indicate the error structure used (n: normal errors, b: binomial errors, qb: quasibinomial). * Blood meal rate: proportion of females which took a blood meal.

| **Variable of interest** | **Resp. variable** | **Model Nb.** | **N** | **Maximal model** | **Minimal model** | **Rsubroutine** |
| --- | --- | --- | --- | --- | --- | --- |
| **Experiment 1** |  |  |  |  |  |  |
| Survival rate | Longevity data | 1 | 586 | Glyph._concertation*Glyph._type | Glyph._concertation | coxph |
| Dev time | day | 2 | 504 | Glyph._concertation*Glyph._type *Sex | Sex | glm[n] |
| Wing size | mm | 3 | 269 | Glyph._concertation*Glyph._type *Sex | Sex | glm[n] |
| Blood meal rate* | Blood fed or not | 4 | 150 | Glyph._concertation*Glyph._type + (1/Bird) | 1 + (1/Bird) | glmer[b] |
| Blood meal size | hm | 5 | 119 | Glyph._concertation*Glyph._type + (1/Bird) | 1 + (1/Bird) | lmer[n] |
| Number of eggs | eggs no | 6 | 74 | Glyph._concertation*Glyph._type *hm + (1/Bird) | hm + (1/Bird) | lmer[n] |
| Oocyst burden | oocyst no | 7 | 104 | Glyph._concertation*Glyph._type *hm + (1/Bird) | hm + (1/Bird) | lmer[n] |
| **Experiment 2** |  |  |  |  |  |  |
| Survival rate | Longevity data | 8 | 618 | Glyph._expostion*Food_treatment | 1 | coxph |
| Dev time | day | 9 | 445 | Glyph._expostion*Food_treatment*Sex | Glyph._expostion*Food_treatment + Sex | glm[n] |
| Wing size | mm | 10 | 312 | Glyph._expostion*Food_treatment*Sex | Food_treatment + Sex | glm[n] |
| Blood meal rate* | Blood fed or not | 11 | 120 | Glyph._expostion*Food_treatment + (1/Bird) | 1 + (1/Bird) | glmer[b] |
| Blood meal size | mg | 12 | 99 | Glyph._expostion*Food_treatment + (1/Bird) | 1 + (1/Bird) | lmer[n] |
| Number of eggs | eggs no | 13 | 25 | Glyph._expostion*Food_treatment*hm + (1/Bird) | hm + (1/Bird) | lmer[n] |
| Infection prevalence | Uninfected = 0, Infected = 1 | 14 | 74 | Glyph._expostion*Food_treatment*hm + (1/Bird) | Glyph._expostion*Food_treatment + (1/Bird) | glmer[b] |
| Oocyst burden | oocyst no | 15 | 64 | Glyph._expostion*Food_treatment*hm + (1/Bird) | hm + (1/Bird) | lmer[n] |


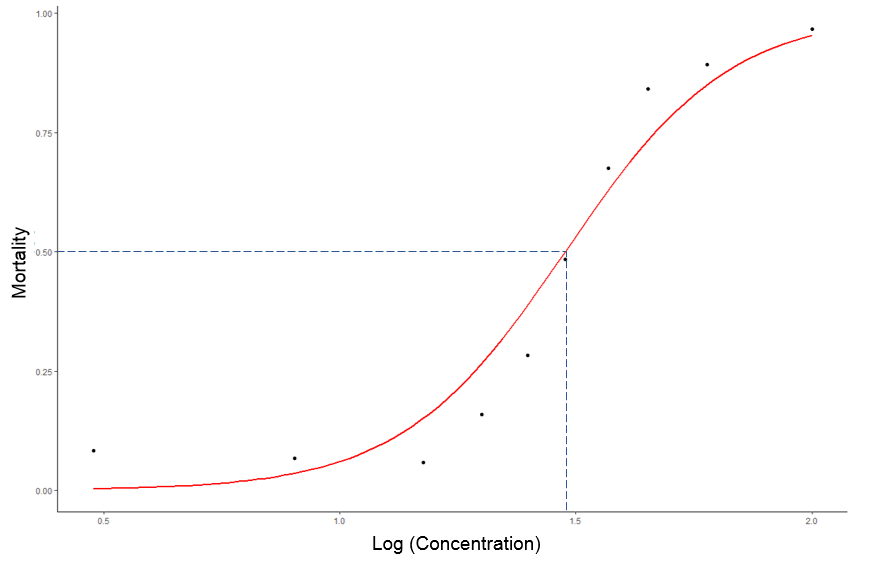


**Figure S1. Dose-response curves of mortality of mosquito larvae after imidacloprid exposure.**

To determine the susceptibility of the mosquito lineage used in our study to imidacloprid and to identify the concentration that kills 50% of the population (LC50), 10 concentrations of imidacloprid were used (3, 8, 15, 20, 25, 30, 37, 45, 60, 100 μg/L). For each concentration, 20 larvae (24-h old) were haphazardly collected and put inside a plastic tank (7.5 x 10.5 x 4.5 cm) filled with 120 mL of the imidacloprid solution and 30 mg of food (which corresponded to 6 mL of solution per larvae and 0.5mg of food per larvae per days). All concentrations of imidacloprid were realized from a stock solution of 2 mg/L, prepared with solid imidacloprid (98 % purity, provided by Sigma-Aldrich, Switzerland) dissolved in mineral water. For each concentration, 6 replicates were made. The mortality was determined after 72-h of exposure.
